# Interethnic Differences in Antigen-Presenting Cell Activation and TLR Responses in Malian Children during *Plasmodium falciparum* Malaria

**DOI:** 10.1371/journal.pone.0018319

**Published:** 2011-03-31

**Authors:** Charles Arama, Pablo Giusti, Stéphanie Boström, Victor Dara, Boubacar Traore, Amagana Dolo, Ogobara Doumbo, Stefania Varani, Marita Troye-Blomberg

**Affiliations:** 1 Department of Immunology, Wenner-Gren Institute, Stockholm University, Stockholm, Sweden; 2 Malaria Research and Training Centre, Faculty of Medicine, Pharmacy and Dentistry, University of Bamako, Bamako, Mali; 3 Department of Laboratory Medicine, Division of Clinical Microbiology, Karolinska University Hospital, Huddinge, Sweden; 4 Department of Haematology and Clinical Oncology, “L. and A. Seragnoli”, University of Bologna, Bologna, Italy; University of Palermo, Italy

## Abstract

The Fulani ethnic group from West Africa is relatively better protected against *Plasmodium falciparum* malaria as compared to other sympatric ethnic groups, such as the Dogon. However, the mechanisms behind this lower susceptibility to malaria are largely unknown, particularly those concerning innate immunity. Antigen-presenting cells (APCs), and in particular dendritic cells (DCs) are important components of the innate and adaptive immune systems. Therefore, in this study we investigated whether APCs obtained from Fulani and Dogon children exhibited differences in terms of activation status and toll-like receptor (TLR) responses during malaria infection. Lower frequency and increased activation was observed in circulating plasmacytoid DCs and BDCA-3^+^ myeloid DCs of infected Fulani as compared to their uninfected counterparts. Conversely, a higher frequency and reduced activation was observed in the same DC subsets obtained from peripheral blood of *P. falciparum*-infected Dogon children as compared to their uninfected peers. Moreover, infected individuals of both ethnic groups exhibited higher percentages of both classical and inflammatory monocytes that were less activated as compared to their non-infected counterparts. In line with APC impairment during malaria infection, TLR4, TLR7 and TLR9 responses were strongly inhibited by *P. falciparum* infection in Dogon children, while no such TLR inhibition was observed in the Fulani children. Strikingly, the TLR-induced IFN-γ release was completely abolished in the Dogon undergoing infection while no difference was seen within infected and non-infected Fulani. Thus, *P. falciparum* infection is associated with altered activation status of important APC subsets and strongly inhibited TLR responses in peripheral blood of Dogon children. In contrast, *P. falciparum* induces DC activation and does not affect the innate response to specific TLR ligands in Fulani children. These findings suggest that DCs and TLR signalling may be of importance for the protective immunity against malaria observed in the Fulani.

## Introduction

Half of the world's population remains at risk of contracting malaria. During 2008 malaria was estimated to be responsible for 767.000 deaths in Africa alone, mostly in children below the age of 5 [1]. Previous studies performed in a rural area in Mali have shown different susceptibility to *Plasmodium falciparum* (*P. falciparum*) infection between two ethnic groups; the Fulani and the Dogon. These populations live under similar social, cultural and geographic conditions and are exposed to identical malaria pressure [2]. However, the Fulani show less clinical symptoms of malaria, and parasites are less frequently detected in their blood [3], [4]. In addition, they exhibit higher titres of *P. falciparum*-specific IgG subclasses [3], and IgM antibodies [4], [5]. Recent evidence shows lower numbers of regulatory T cells [6] and more potent early IFN-γ responses against the parasite [7] in Fulani as compared to other sympatric ethnic groups, suggesting that these immunological phenomena can lead to more robust anti-malarial immunity. Nevertheless, little is known about the functionality of innate immunity in Fulani and sympatric ethnic groups and how this branch of immunity influences susceptibility to malaria infection.

Innate immunity has two important functions in the defence against the parasite. First, it acts to inhibit rapid parasite growth thereby limiting the onset of malaria pathology. Second, it is crucial for the development of parasite-specific adaptive immunity [8]. Antigen-presenting cells (APCs), and in particular dendritic cells (DCs) are important components of the innate and adaptive branches of the immune system. In humans, two main populations of DCs have been described in peripheral blood; the CD11c^−^, CD123^+^, blood dendritic cell antigen (BDCA)-2^+^ plasmacytoid DCs (pDCs) and the CD11c^+^, CD123^−^ myeloid DCs (mDCs). The latter can be further divided into three subtypes; the BDCA-1^+^ DCs, the BDCA-3^+^ DCs and the CD16^+^ DCs [9]–[11].

It has been shown that monocyte/macrophage- and mDC functions are impaired *in vitro* by the malaria parasite or by products derived from infection [12]–[14]. Another study has shown that HLA-DR levels are decreased on circulating DCs of *P. falciparum*-infected children as compared to healthy controls [15]. In addition, higher numbers of circulating BDCA-3^+^ mDCs have been detected in the blood of severely infected children combined with reduced immunostimulatory ability. This indicates that malaria may impair mDC functionality and suggests that the BDCA-3^+^ DC subset may play a crucial role in the immune response against the parasite in humans [16], [17].

*P. falciparum* also modulates pDC responses *in vitro* by inducing increased secretion of interferon (IFN)-α [18]. Daily injection of this cytokine protects against severe malaria caused by *P. berghei* in mice [19] and higher plasma levels of IFN-α have been associated with milder *P. falciparum* malaria episodes in humans [20]. However, while mDCs are crucial in triggering an effective T-cell response during malaria infection, pDCs are not required for the induction of malaria parasite-specific responses as recently shown in murine models of experimental infection [21], [22].

Evidence demonstrates a fundamental role for monocytes in activation of NK cells [23] and in differentiation of regulatory T cells [24] upon *P. falciparum* stimulation *in vitro*. Nevertheless, the role of this cell subset during the infection in the natural host is only partially understood. Monocytes can trigger adaptive immune responses and contribute to parasite clearance through antibody-dependent cellular inhibition and phagocytosis [25]. Two major monocyte subpopulations have been described [26]; one classical (CD14^+^CD16^−^) and one inflammatory (CD14^+^CD16^+^) [27]; the latter has been found to be increased during acute uncomplicated malaria [28], [29]. A recent study reported that the levels of CD16/Fcγ receptor IIIA on CD16^+^ monocytes were higher in children with severe *P. falciparum* anaemia, which could enhance erythrophagocytosis and TNF-α production in this cell subset [30].

APCs interact with pathogens through specialized receptors termed pattern-recognition receptors (PRRs) that have evolved to detect danger signals such as pathogen-associated molecular patterns (PAMPs) [31]. A well-studied family of PRRs are the toll-like receptors (TLRs) [32], which are expressed differently in different APC populations. The monocyte and mDC subsets express mainly TLR1 through TLR6, while TLR7 and TLR9 are expressed in pDCs [33], [34]. Recent evidence suggests that TLRs are central mediators of pro-inflammatory responses during malaria [35]–[37]. For example, it is known that *Plasmodium* DNA bound to either malaria pigment [37] or protein components [38], activates the TLR9 pathway and that *P. falciparum* glycosylphosphatidylinositol (GPI) activates TLR2 [39]. However, the role of TLRs in the development of immunity to malaria and in the pathology of malaria is not well understood.

In this study, we evaluated the activation status of different DC and monocyte subpopulations in peripheral blood of Fulani and Dogon children with or without *P. falciparum* malaria infection in an endemic area of Mali. In addition, the impact of *P. falciparum* on the activation of APCs was examined by stimulating peripheral blood mononuclear cells (PBMCs) with specific TLR ligands. LPS was employed to activate TLR4 on monocytes and mDC subsets while imiquimod and CpG-A ODN were used to selectively stimulate TLR7 and TLR9, respectively, which are expressed in pDCs [9], [10], [40].

## Results

### Study subject characteristics

The mean age of the Dogon children was 6.36 years for uninfected and 6.70 years for the infected ones. In the Fulani children, the mean age was 6.44 years for uninfected and 4.50 years for the infected ones. The infected Fulani were younger than the infected Dogon (Table 1). During *P. falciparum* infection, the mean temperature was significantly higher in the infected Dogon (37.74°C) as compared to infected Fulani (36.71°C, p≤0.05). The mean parasite density was 18758 [150-122000] asexual parasites/µl for Fulani and 13845 [575-48625] asexual parasites/µl for the Dogon.

**Table 1:** Baseline characteristics of the study population.

n = 77	Mean age (Year)	Mean Hb level (g/dL)	Mean body weight (Kg)	Parasitaemia Mean [min-max] (parasites/µL)	Mean axillary temperature (°C)
Uninfected Dogon	6.36	10.36	19.25	-	36.43
Infected Dogon	6.70	9.80	20.15	13845 [575-48625]	37.74
Uninfected Fulani	6.44	9.78	17	-	36.71
Infected Fulani	4.50	9.36	12.93	18758 [150-122000]	36.81
P values	*DI-FI *F-FI	NS	*DI-FI *F-FI	NS	*D-DI *DI-FI

Note. D: Uninfected Dogon; DI: Infected Dogon; F: Uninfected Fulani; FI: Infected Fulani;

*p value≤0.05 Fisher exact test (Anova); NS: not significant.

### Interethnic and intraethnic differences in the frequency and immunophenotype of blood DCs during *P. falciparum* infection

Circulating DCs are defined as leukocyte lineage negative and HLA-DR positive mononuclear cells [41]. Blood DCs were divided into four different subsets by employing monoclonal antibodies against the surface markers BDCA-1, BDCA-2, BDCA-3 and CD16 after gating lineage negative PBMCs. The activation status of each subset was assessed by measuring HLA-DR and CD86 levels.

**Figure 1:**
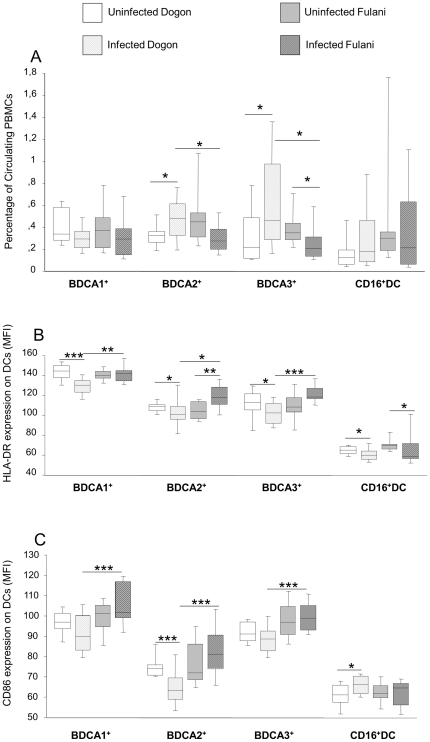
Interethnic and intraethnic differences in the frequency and activation markers of blood DCs. PBMCs from the study participants were stained with monoclonal antibodies for subsequent flow cytometric analysis using a FACSCalibur. The samples were categorised as uninfected Dogon (n = 20), infected Dogon (n = 20), uninfected Fulani (n = 13) and infected Fulani (n = 10). An increase was observed of BDCA2^+^ and BDCA3^+^ cells in the circulation of infected Dogon whereas the opposite was seen for the Fulani (Figure 1A). The expression of activation marker HLA-DR (Figure 1B) and CD86 (Figure 1C) was lower in BDCA2^+^ and BDCA3^+^ cells of the Dogon when undergoing infection while it was increased in the Fulani. It is known that DCs travel to the lymph nodes upon activation. Therefore, it is possible that the lower numbers seen in the infected Fulani is a consequence of activation. The boxplots illustrate the medians and the 25^th^ and 75^th^ quartile and the whiskers represent the 10% and 90% percentiles. Data were analyzed by Mann-Whitney rank sum test. *; p≤0.05. **;p<0.01. ***; p<0.001.

The infected Dogon exhibited significantly higher frequency of circulating BDCA-2^+^ (p = 0.034) and BDCA-3^+^ (p = 0.016) DCs than their uninfected peers (Figure 1A). Conversely, the infected Fulani had a slightly reduced frequency of BDCA-2^+^ (p = 0.060) and BDCA-3^+^ (p = 0.038) DCs as compared to their uninfected peers. When comparing only the infected children from each ethnic group, the percentage of circulating BDCA-2^+^ and BDCA-3^+^ DCs was significantly lower in Fulani as compared to Dogon (p = 0.04 and p = 0.01, respectively). No interethnic differences were observed between the uninfected children from each group. A similar trend was observed when evaluating absolute numbers of BDCA-2^+^ and BDCA-3^+^ DCs/ml of blood although differences were not significant (data not shown). The percentage and absolute numbers of circulating CD16^+^ and BDCA-1^+^ DCs did not show any inter- or intra-ethnic difference (Figure 1A and data not shown).

When examining DC-activation status, we observed that expression levels of the activation marker HLA-DR was significantly lower on all examined DC subsets in the infected Dogon as compared to uninfected peers (Figure 1B). In addition, the Dogon children undergoing infection had a decreased expression of CD86 on BDCA-2^+^ DCs (Figure 1C) as compared to their uninfected peers (p = 0.0003). Conversely, the infected Fulani children exhibited significantly increased levels of HLA-DR on pDCs (p = 0.009) and slightly increased levels of HLA-DR on BDCA-3^+^ mDCs (p = 0.057) as compared to uninfected peers. In addition, expression levels of HLA-DR were reduced on CD16^+^ mDCs of both Dogon and Fulani children undergoing infection as compared to their uninfected peers.

When comparing children undergoing infection from each ethnic group, the Fulani exhibited higher levels of HLA-DR and CD86 on the BDCA-1^+^, BDCA-2^+^ and BDCA-3^+^ DCs but not on CD16^+^ DCs as compared to the infected Dogon children. Thus, interethnic differences were observed in terms of blood DC activation status upon malaria and all circulating DC subpopulations excluding CD16^+^ DCs were significantly more mature in Fulani than in Dogon children during *P. falciparum* infection. In order to evaluate if these differences could be dependent on parasite burdens, we correlated levels of parasitaemia with HLA-DR expression levels on the affected DC subpopulations from each study participant. No significant correlation was observed in Dogon and Fulani children undergoing *P. falciparum* infection. For BDCA-1^+^ DCs the results of the correlation tests were Rho = −0.38 (p = 0.106) for Dogon children and Rho = 0.20 (p = 0.72) for Fulani children. For BDCA-2^+^ DCs the results were Rho = −0.16 (p = 0.49) for Dogon children and Rho = 0.20 (p = 0.72) for Fulani children. For BDCA-3^+^ DCs the results were Rho = −0.28 (p = 0.22) for infected Dogon children and Rho = −0.40 (p = 0.48) for infected Fulani children. This suggests that the differences observed in the DC subpopulations could not be correlated to the differences in parasite burden between the populations.

### Interethnic and intraethnic differences in monocyte frequency and activation status during *P. falciparum* infection

Given the importance of monocytes in inflammation and clearance of parasites, we investigated the classical (CD14^+^CD16^−^) and inflammatory (CD14^+^CD16^+^) monocytic subsets during *P. falciparum* infection in the children participating in the study.

**Figure 2:**
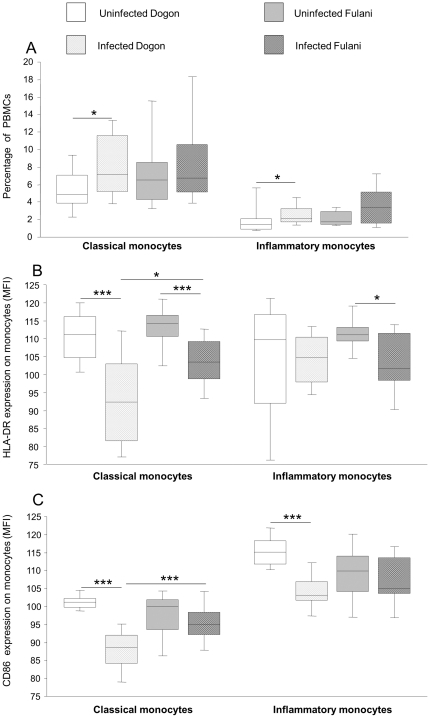
Interethnic and intraethnic differences in the frequency and activation markers of circulating monocytes. PBMCs were stained with monoclonal antibodies and subsequently analyzed using FACSCalibur. The samples were categorized as uninfected Dogon (n = 20), infected Dogon (n = 20), uninfected Fulani (n = 13) and infected Fulani (n = 10). An increase of circulating monocytes was observed for both groups although only statistically significant for the Dogon (Figure 2A). There was a decreased expression of HLA-DR (Figure 2B) in classical monocytes for both ethnic groups. The levels of HLA-DR were also suppressed in the inflammatory monocytes although not statistically significant for the Dogon. The expression of CD86 (Figure 2C) was decreased on both monocytic subsets although only statistically significant for the Dogon classical monocytes from both Dogon and Fulani. Although the levels of activation markers seem similarly regulated in both groups the classical monocytes of the Fulani exhibit a pattern of higher activation when undergoing infection than the Dogon when infected. The boxplots illustrate the medians and the 25^th^ and 75^th^ quartile and the whiskers represent the 10% and 90% percentiles. Data were analyzed by Mann-Whitney rank sum test. *; p≤0.05. ***; p<0.001.

A significantly increased proportion of both classical (p = 0.02) and inflammatory (p = 0.02) monocytes was observed in Dogon children undergoing infection as compared to their uninfected peers. A similar trend, although not significant, was found between infected and non-infected Fulani (Figure 2A).

When examining the activation status of circulating monocytes, a significantly decreased expression of HLA-DR was observed on classical monocytes from both Dogon and Fulani groups undergoing infection as compared to their uninfected peers (p = 0.0001 and p = 0.004, respectively; Figure 2B). A decreased expression of HLA-DR was also observed in inflammatory monocytes of Fulani (p = 0.02) but not of Dogon children (p = 0.41) undergoing infection as compared to their uninfected peers. Finally, in infected Dogon children the expression of the co-stimulatory marker CD86 was also significantly impaired on classical and inflammatory monocytes as compared to uninfected peers (Figure 2C). When comparing children undergoing infection from each ethnic group, the Fulani exhibited higher levels of HLA-DR and CD86 on classical but not on inflammatory monocytes as compared to Dogon children. Thus, interethnic differences were observed in classical monocytes during malaria infection and these cells were significantly more activated in infected Fulani than in infected Dogon children.

### *P. falciparum* infection impairs cytokine responses upon TLR stimulation in Dogon but not in Fulani children

The ability of APCs to secrete cytokines upon TLR stimulation was investigated by stimulating PBMCs with ligands for TLR4, TLR7 and TLR9, i.e. LPS, imiquimod and CpG-A, respectively.

**Figure 3:**
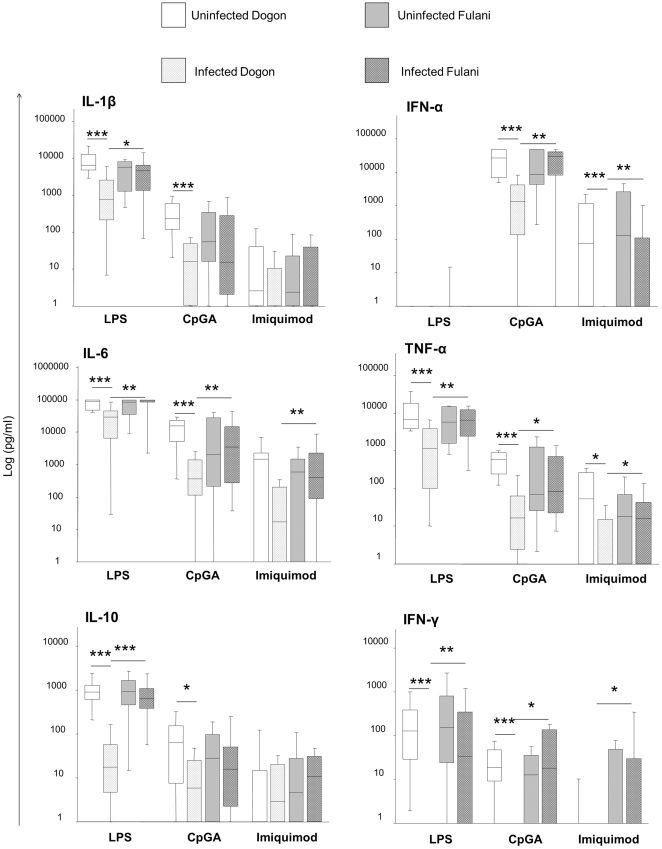
TLR-induced cytokine responses in PBMCs from Dogon and Fulani children with or without *P. falciparum* infection. PBMCs (1 million/ml) were stimulated or not with LPS (100 ng/ml), CpG-A (3 µg/ml) or imiquimod (1 µg/ml). After 16 hours, supernatants were collected and analysed for the presence of cytokines. The samples were categorized as uninfected Dogon (n = 20), infected Dogon (n = 20), uninfected Fulani (n = 14) and infected Fulani (n = 23). The levels of IL-1β, IL-6, IL-10, TNF-α and INF-γ were severely supressed in the infected Dogon as compared to their uninfected peers when the cells had been stimulated with either LPS or CpGA. As a result, the levels of the infected Fulani were higher than infected Dogon for all cytokines except for IFN-α when stimulated with LPS and except for IL-1β and IL-10 when stimulated with CpG. Similarly, when stimulated with imiquimod cells of the infected Fulani secreted higher levels of IL-6, IFN-α, TNF-α and INF-γ than the infected Dogon due to the suppressed secretion of Dogon when undergoing infection. Data presented as box plots illustrate the medians and the 25^th^ and 75^th^ quartile and the whiskers represent the 10% and 90% percentiles. Data were analyzed using Mann-Whitney rank sum test. *; p≤0.05. **; p<0.01. ***; p<0.001.

In Dogon, the TLR4 pathway was strongly impaired during *P. falciparum* infection, as shown by inhibited production of IL-1β, IL-6, IL-10, TNF-α and IFN-γ by PBMCs upon LPS stimulation (Figure 3). Similarly, release of IL-1β, IL-6, IL-10, TNF-α, IFN-α and IFN-γ was inhibited in infected Dogon as compared to uninfected peers when PBMCs were treated with a TLR9 ligand. Also TLR7 responses were impaired upon *P. falciparum* infection in Dogon children and IFN-α and TNF-α levels were lower in PBMCs stimulated with imiquimod in infected Dogon as compared to their uninfected peers.

Conversely, no differences were observed in cytokine release between PBMCs obtained from infected and uninfected Fulani upon TLR4, TLR7 and TLR9 stimulation (Figure 3). As compared to infected Dogon children, the infected Fulani secreted significantly higher levels of IL-1β, IL-6, IL-10, TNF-α and IFN-γ upon TLR4 stimulation and higher levels of IL-6, IFN-α, TNF-α and IFN-γ when PBMCs were stimulated with TLR7 or TLR9 ligands.

Taken together, these results indicate that *P. falciparum* infection impaired TLR4-, TLR7-, and TLR9-induced cytokine responses in the Dogon children, while such responses were unaffected in the Fulani children. As a consequence, the levels of most cytokines that were released upon TLR stimulation were higher in Fulani than Dogon children undergoing *P. falciparum* infection.

**Figure 4:**
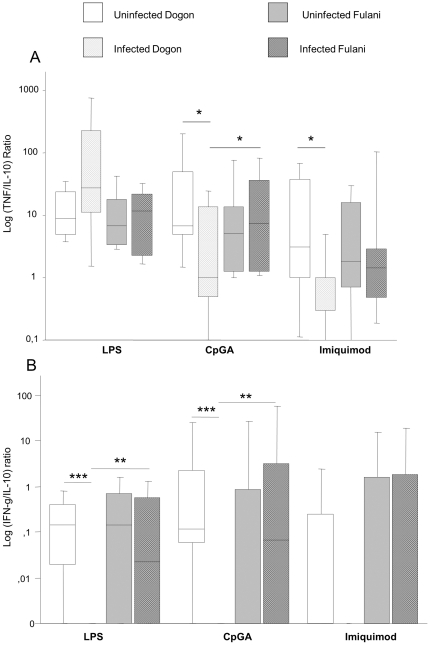
Interethnic and intraethnic differences in the TNF-α/IL-10 and IFN-γ/IL-10 ratios upon TLR stimulation of PBMCs. PBMCs (1 million/ml) were stimulated or not with LPS (100 ng/ml), CpG-A (3 µg/ml) or imiquimod (1 µg/ml). After 16 hours, supernatants were collected, cytokine levels were analyzed and the TNF-α/IL-10 (Figure 4A) and IFN-γ/IL-10 (Figure 4B) ratios were calculated. The samples from 77 children were categorized as uninfected Dogon (n = 20), infected Dogon (n = 20), uninfected Fulani (n = 23) and infected Fulani (n = 14). The TNF-α/IL-10 ratio (Figure 4A) was significantly lower in the infected Dogon as compared to their uninfected peers when stimulated with CpG or imiquimod. This resulted in significantly higher TNF-α/IL-10 ratio of infected Fulani as compared to the infected Dogon. Due to the severe suppression of IFN-γ the IFN-γ/IL-10 ratios (Figure 4B) were severely suppressed in the LPS and CpG stimulated cells of the Dogon and therefore higher in the infected Fulani than in the infected Dogon. The boxplots illustrate the medians and the 25^th^ and 75^th^ quartile and the whiskers represent the 10% and 90% percentiles. Data were analyzed using Mann-Whitney rank sum test. *; p≤0.05. **; p<0.01. ***; p<0.001.

To further analyse the clinical relevance of the cytokines secreted by the PBMCs from Dogon and Fulani children when exposed to *P. falciparum* infection, the ratios of TNF-α/IL10 and IFN-γ/IL-10 were calculated. The TNF-α/IL10 ratio was significantly decreased upon *P. falciparum* infection in Dogon children when PBMCs were stimulated with TLR7 (p = 0.02) and TLR9 (p = 0.01) ligands, indicating an impaired pro-inflammatory activity in these specific TLR pathways upon *P. falciparum* infection in this ethnic group (Figure 4A). Conversely, the TNF-α/IL10 ratio was slightly increased upon *P. falciparum* infection in Dogon children when PBMCs were stimulated with LPS (p = 0.08). This might indicate that the TLR4 signalling pathway may be less affected than the TLR7 and TLR9 signalling in Dogon undergoing infection. This ratio was not affected in infected Fulani as compared to uninfected peers upon TLR4, TLR7 and TLR9 stimulation. When comparing children undergoing infection from each ethnic group, the TNF-α/IL-10 ratio was higher in Fulani than Dogon children when cells were stimulated with TLR9 ligands (p = 0.04), suggesting a significantly higher proinflammatory activity induced by TLR9 ligation in Fulani than Dogon children undergoing *P. falciparum* infection. Similarly, the ratio of IFN-γ/IL-10 was decreased in the infected Dogon children as compared to their uninfected peers upon stimulation with TLR4 (p<0.0001) and TLR9 (p<0.0001; Figure 4B). When comparing PBMCs obtained from children undergoing infection from each ethnic group, the IFN-γ/IL-10 ratio was significantly higher in Fulani than in Dogon upon stimulation with TLR4 and TLR9 ligands (p = 0.002 and p = 0.0051; respectively). The same patterns were observed for cells stimulated with TLR7 although the data did not reach statistical significance. Thus, these data confirm that, during *P. falciparum* malaria infection, PBMCs from Fulani children exhibit a higher pro-inflammatory profile upon TLR stimulation as compared to the Dogon children.

## Discussion

In this study, we investigated certain aspects of the innate immunity to malaria in children from two different ethnic groups that live in a malaria endemic area in Mali and exhibit different susceptibility to *P. falciparum* infection. The impact of ethnicity on allele and genotype frequency for innate soluble factors, such as IL-4 and IL-10, has been previously established in asymptomatic individuals belonging to Dogon and Fulani's groups in Mali [42]. However, functional studies focusing on the role of *P. falciparum* in affecting innate responses in these two ethnic groups are lacking.

Our findings indicate that *P. falciparum* infection inhibited the expression of HLA-DR in mDCs and pDCs in Dogon children. These results are in line with previous findings showing reduced expression of HLA-DR on mDCs of Kenyan children during acute malaria [15]. HLA class II expression on DCs is fundamental for presenting antigens to T cells and inducing their activation [43]. Higher numbers of circulating BDCA-3^+^ mDCs have been detected in blood of severely infected children [16]. Similarly, we observed that the frequency of this mDC subset was increased in peripheral blood of Dogon children undergoing malaria infection as compared to uninfected peers. It has also been previously shown that APCs from children with severe malaria exhibit lower immunostimulatory abilities than those of healthy children [16]. Thus, the reduced expression of HLA-DR on the surface of DCs in Dogon children undergoing malaria infection may indicate impaired DC functionality, which in turn, may contribute to impaired cell-mediated responses.

Conversely, higher activation levels were observed in blood pDCs and BDCA-3^+^ mDCs of infected Fulani as compared to uninfected children belonging to the same ethnic group. It is known that DC activation is coupled to increased cell migration to lymphoid organs [41]. Therefore, activation of these DC subsets may trigger their migration to the spleen and other lymphoid organs and thereby account for the lower frequency of pDCs, and BDCA-3^+^ mDCs observed in the peripheral blood of infected Fulani. However, the absolute numbers of circulating DCs were only slightly affected by malaria infection in Fulani. Therefore, the reduced frequency of these DC subsets in infected subjects may also be explained by a relative increase in some other leukocyte population. In fact, it is known that the Fulani have a higher spleen rate than other sympatric ethnic groups [2], [5], [44], [45] that could be coupled to an increased number of T- and B-cell subsets in circulation. Thus, it is possible that circulating BDCA-3^+^ mDCs and pDCs become activated and migrate to secondary lymphoid organs such as the spleen to a higher extent in the Fulani than in the Dogon during malaria infection.

Recent evidence suggests that BDCA-3^+^ DCs in humans exhibit superior capacity in stimulating cytotoxic T-cell (CTL) responses. These cells may be the counterpart of murine CD8-α^+^ DCs [11], [46], which are known to cross-present parasite antigens and mediate effective CTL responses against blood stage *P. berghei* in a murine model of malaria [47]. In addition, pDCs are known to play a crucial role in triggering humoral immunity against viruses [48], [49]. Thus, the specific activation of these DC subsets observed in the Fulani, but not in the Dogon, during *P. falciparum* infection may contribute to more robust anti-malarial cellular IFN-γ responses [7] and antibody responses [3], [5], [44], [50] that have been observed in the Fulani as compared to other sympatric ethnic groups.

The frequencies of both classical and inflammatory monocytes were increased both in Dogon and Fulani children upon *P. falciparum* infection. These results are in accordance with previous findings in Thai-Burmese patients where patients with acute malaria exhibited a higher frequency of inflammatory monocytes in peripheral blood [28]. We also found that *P. falciparum* infection impaired monocyte immunophenotypes, as reflected by the reduced expression of activation markers on classical and inflammatory monocytes in both Fulani and Dogon children, suggesting that *P. falciparum* alters the functionality of these cells in both ethnic groups. However, interethnic differences were observed during malaria infection as classical monocytes were significantly more activated in infected Fulani than in infected Dogon children.

To further evaluate the innate response in the Fulani and Dogon children during malaria infection, we investigated cytokine release by PBMCs after stimulation with specific TLR ligands. Cytokines are crucial in the pathogenesis of malaria and the balance between pro- and anti- inflammatory cytokines plays a central role in the clinical outcome of this infection [51], [52]. In this context, we report that, in Dogon children, TLR4, TLR7 and TLR9 pathways were impaired by *P. falciparum* infection while such TLR inhibition was not observed in the Fulani children. This is in line with the impairment of DC phenotype that was observed in Dogon but not Fulani children upon malaria infection. In humans, TLR7 and TLR9 are mainly expressed in pDCs and it has been established that TLR9 is involved in recognition of malarial products [37], [38], [53]. It is also known that TLR7 and TLR9 signalling mediates robust induction of type I IFNs, increased cross presentation of antigens and strong CTL responses [54], [55]. In our study, impairment has been specifically observed in the pro-inflammatory activity of these two TLR pathways upon *P. falciparum* infection in Dogon children, but not in the Fulani ethnic group.

In general, the importance of TLRs in malaria is underlined by the fact that TLR4 polymorphisms can predispose to severe malaria [56] and that polymorphisms in both TLR4 and TLR9 may play a role in the severity of pregnancy associated malaria [57]. Contradictory findings however, have been reported about the effect of malaria infection on TLR signalling. Enhanced pro-inflammatory cytokine production has been shown in response to TLR1/2, TLR4, TLR7/8 and TLR9 stimulation in PBMCs obtained from volunteers undergoing experimental malaria [58] or patients with natural infection [59], while others have suggested that malaria has a suppressive effect on TLR signalling, possibly through cross-tolerance following recognition of *Plasmodium* antigens by TLRs [60]. In partial agreement with the latter study, we observed that *P. falciparum* infection suppresses TLR responses in Dogon children while no effects were observed in the Fulani children. There may be several reasons for the discrepancies seen in these studies. Firstly, both the studies by McCall et *al*. and Franklin et *al*. were performed in adults while, in our study, only children were included. Secondly, these studies were performed in malaria naïve adults at the earliest stages of *P. falciparum* infection [58] and in an area with low transmission of malaria [61], respectively. Conversely, our study area is mesoendemic with high transmission during rainy seasons. As this work was performed at the end of the rainy season, the children included in our study should have experienced at least 2 episodes of *P. falciparum* infection during the same season as estimated by previous observations in the same area (Arama C. unpublished results). Thus, malaria infection may have a biphasic influence on the innate immune system, initially inducing TLR priming and then, in case of frequent exposure to the parasite, tolerance will ensue. Interestingly, such a suppressive phase was observed in Dogon but not in the children belonging to the Fulani ethnic group.

Moreover, we observed higher release of IFN-γ by PBMCs of infected Fulani children as compared to infected Dogon upon TLR stimulation. This is in line with recent findings indicating that PBMCs from Fulani specifically stimulated with *P. falciparum in vitro* release higher amount of IFN-γ than Dogon [7]. Studies in both humans and mice indicate that the magnitude of an early IFN-γ response is a crucial determinant of the outcome of malaria infection [23], [62]. Our findings suggest that the impaired innate IFN-γ responses upon TLR stimulation in infected Dogon as compared to infected Fulani may be one of the underlying causes for the relative susceptibility to malaria in the Dogon ethnic group as compared to the Fulani.

It is possible that APC dysfunction could be dependent on the levels of parasitaemia in an infected individual. This suggests that the APC alteration observed in Dogon individuals upon *P. falciparum* infection merely depends on differences in parasitaemias in this ethnic group as compared to Fulani. However, we did not observe any significant correlation between parasitaemia and HLA-DR expression levels on distinct APC subsets in infected children. Therefore, differences in levels of parasitaemia do not appear to account for the APC dysfunction observed in Dogon children as compared to Fulani in our cohort.

In conclusion, in this study we found that *P. falciparum* infection impairs the phenotype of blood DCs and alters TLR responses of PBMCs from Dogon but not Fulani children. In particular, malaria infection induces differential innate IFN-γ release in the two ethnic groups. These findings suggest that activation of certain DC subsets and additional mechanisms triggering cytokine release by APCs through TLR activation may represent immunological correlates of protection against *P. falciparum* malaria. However, the prospective evaluation of a larger cohort of patients is required to confirm our results and to analyze the potential occurrence of distinct TLR polymorphisms in these two ethnic groups. The understanding of innate immune responses to *P. falciparum* in naturally exposed children may yield important insights in the development of immunity to malaria with particular regard to rational vaccine development.

## Materials and Methods

### Ethics statement

Ethical approval was obtained from the institutional review boards of the University of Bamako, Mali (N°08_64/FMPOS) and by the Swedish ethical research committee (03-536). Before the inclusion of each child in the study, the community leaders' permission and individual consent form were obtained from the parents of each child.

### Study population

The study was conducted in a rural area of the Dogon valley of Mali where malaria is mesoendemic with intense transmission during the rainy season. The study area and the study population have been described in details elsewhere [2]. Children between 2 and 10 years of age belonging to either the Fulani or the Dogon ethnic group were included in the study. Forty children from the Dogon population and 37 from the Fulani were recruited. The study included healthy children and infected children that were classified as uncomplicated malaria. A thick blood smear was performed in all subjects included in the study. The slides were stained in 3% Giemsa and examined for the presence of *P. falciparum* parasites. Presence of malaria infection was defined as a positive thick smear with or without any malaria symptom. Among the Dogon, 20 children were undergoing malaria infection and 20 children were healthy, while among Fulani 14 children were suffering from malaria and 23 were uninfected. Unfortunately not all slides could be recovered from the field study so we only have parasitaemias for eight of the Fulani and nineteen of the Dogon children.

### Blood collection and cell preparation

Venous whole blood samples were collected from healthy and infected children of both ethnicities. From each volunteer, 6 ml of peripheral blood was collected in heparin tubes (BD Vacutainer® Plasma Tube, Franklin Lakes, NJ, USA) and were transported to the Malaria Research and Training Centre Laboratory in Bandiagara, north-east of Mali, for further experiments. PBMCs were isolated by density gradient centrifugation using Ficoll-Paque (GE Healthcare, Uppsala, Sweden) and used for APC immunophenotyping and TLR stimulation.

### Immunophenotype

Purified PBMCs were fixed and frozen immediately after purification as previously described [63]. Briefly, cells were resuspended in PBS to a concentration of 2 million cells/ml and treated with DNAse (Sigma-Aldrich®, Steinheim, Germany) for 5 minutes at 37°C at a concentration of 666 units/ml. Cells were then washed and resuspended in 3 ml of prewarmed (37°C) 4% paraformaldehyde (PFA) (Sigma-Aldrich®, Steinheim, Germany), incubated for 5 minutes and then washed in cold PBS +1% foetal bovine serum (FBS, Gibco, Paisley, UK) Finally, cells were resuspended in 1 ml PBS +10% DMSO and frozen at −80°C. For staining, cells were thawed and resuspended in PBS 5 mM EDTA 2% FBS. FcR blocking reagent and mouse monoclonal antibodies conjugated with fluorescein isothiocyanate (FITC) or phycoerythrin (PE), peridinin chlorophyll protein (PerCP) or allophycocyanin were added. Antibodies used for cell-surface staining included those recognizing BDCA-1 (clone AD5-8E7), BDCA-2 (clone AC144), BDCA-3 (clone AD5-14H12), CD3 (BW264/56), CD14 (clone TUK4), CD19 (clone LT19), and mouse isotype controls IgG1 and IgG2a (Miltenyi Biotec, GmbH, Germany) and CD16 (clone B73.1), CD56 (clone NCAM16.2), CD86 (clone 2331 FUN-1), HLA-DR (clone L243 G46-6; Pharmingen, San Diego, CA, USA). The cells were finally resuspended in PBS 1% PFA, acquired and analysed using a FACSCalibur (BD Biosciences, San Diego, CA, USA) and the BD CellQuest™Pro version 5.2.1 software.

### PBMCs stimulation with TLR ligands

PBMCs were resuspended in RPMI supplemented with 10% FCS, 200 mM L-Glutamine, 100 µg/ml streptomycin and 100 U/ml penicillin to a final concentration of 1 million cells/ml. Two hundred µl of cell suspension were distributed into 5 different wells of 96-well flat bottom plate for cell cultures (Sarstedt Inc. Newton, NC, USA). Cells were stimulated with LPS (Sigma-Aldrich® Steinheim, Germany) (100 ng/ml), CpG-A (Metabion, Martinsried, Germany) (3 µg/ml), imiquimod (InvivoGen, San Diego, USA) (1 µg/ml) and unstimulated control. The plates were incubated for 16 hrs at 37°C + 5% CO2 and then the cells and supernatants were collected and stored at −80°C for subsequent cytokine analysis.

### Determination of cytokines in supernatants by cytometric bead array

Levels of IL-1β, IL-6, IL-8, IL-10, IL-12 (p70) and TNF-α in supernatants obtained from stimulated PBMCs were determined by cytometric-bead array (CBA) technology (BD Biosciences, San Diego, CA, USA) and the fluorescent signals were detected by Flow Cytometry according to the manufacturer's recommendation. This sensitive technique allows detection by flow cytometry of multiple cytokines in small quantities of samples. All samples were diluted 1∶10 in Assay Diluent (BD Biosciences, San Diego, CA, USA). Data were acquired with fluorescent activated cell sorter (FACS) Calibur (BD Biosciences, San Diego, CA, USA) and analyzed using CellQuest and FCAP Array software. The lower detection limit for the various cytokines was 4.8, 4.7, 3.2, 3.8, 3.7 and 4.9 pg/ml for IL-1β, IL-6, IL-8, IL-10, IL-12 (p70) and TNF-α, respectively.

### Determination of cytokine levels in supernatants by ELISA

Levels of IFN-α and IFN-γ were measured by conventional sandwich enzyme-linked immunosorbent assay (ELISA) kits using pairs of capture and detection antibodies (Mabtech, Nacka, Sweden). All samples were tested in duplicates according to the manufacturer's recommendation. The optical densities were measured at 405 nm in an ELISA plate reader (VmaxTM Kinetic Microplate Reader, Menlo Park, CA, USA). The assay sensitivity was 2 pg/ml for IFN-α and 7 pg/ml for IFN-γ, respectively.

### Statistical analysis

Statistical significance was determined using non-parametric tests. A Kruskal-Wallis test was employed to analyze the results when more than two groups were involved. The Mann-Whitney U-test was employed to evaluate significance between two unpaired groups when the Kruskal-Wallis test showed a significant difference between the four groups. Linear regression analysis (Spearman rank correlation test) was employed when analysing possible correlations. For all tests p≤0.05 was considered significant and no correction was made for using multiple tests. The data were analyzed using the StatView software.
